# Development and Optimization of a Bromothymol Blue-Based PLA2 Assay Involving POPC-Based Self-Assemblies

**DOI:** 10.3390/ijms25179517

**Published:** 2024-09-01

**Authors:** Shibbir Ahmed Khan, Marc A. Ilies

**Affiliations:** Department of Pharmaceutical Sciences, Moulder Center for Drug Discovery Research, Temple University, School of Pharmacy, 3307 N Broad Street, Philadelphia, PA 19130, USA; shibbir1354@temple.edu

**Keywords:** phospholipase A2, enzymatic assay, pH variation, POPC, liposomes, Triton X-100, mixed micelles, bromothymol blue, PLA2 kinetic study, inhibition study

## Abstract

Phospholipase A2 (PLA2) is a superfamily of phospholipase enzymes that dock at the water/oil interface of phospholipid assemblies, hydrolyzing the ester bond at the sn-2 position. The enzymatic activity of these enzymes differs based on the nature of the substrate, its supramolecular assemblies (micelle, liposomes), and their composition, reflecting the interfacial nature of the PLA2s and requiring assays able to directly quantify this interaction of the enzyme(s) with these supramolecular assemblies. We developed and optimized a simple, universal assay method employing the pH-sensitive indicator dye bromothymol blue (BTB), in which different POPC (3-palmitoyl-2-oleoyl-sn-glycero-1-phosphocholine) self-assemblies (liposomes or mixed micelles with Triton X-100 at different molar ratios) were used to assess the enzymatic activity. We used this assay to perform a comparative analysis of PLA2 kinetics on these supramolecular assemblies and to determine the kinetic parameters of PLA2 isozymes IB and IIA for each supramolecular POPC assembly. This assay is suitable for assessing the inhibition of PLA2s with great accuracy using UV-VIS spectrophotometry, being thus amenable for screening of PLA2 enzymes and their substrates and inhibitors in conditions very similar to physiologic ones.

## 1. Introduction

Phospholipase A2 (PLA2) is a superfamily of lipases that hydrolyze the ester bond at the sn-2 position of the phospholipids, generating a free fatty acid and a lysophospholipid [1,2,3]. The PLA2s are amphiphilic in nature and work only at the water/lipid interface, acting on phospholipid assemblies rather than on isolated single phospholipids [1,2,3].

Enzymatic assays are important for measuring enzyme activity, characterizing new enzymes and assessing their purity and kinetic parameters, determining biological relevance, and screening new activators/inhibitors [1,2,3,4]. With PLA2 assay methods being developed since the 1920s [1,2,3,4], it is important to note that a single assay cannot serve all purposes. Each assay has its advantages and disadvantages; therefore, when selecting a particular assay, it is important to consider the main purpose for its use and its sensitivity and generality [1,2,3,4]. Many PLA2s and related lipases are currently used in a plethora of biological and biotechnological applications due to their wide range of substrate processivity, significant stereo- and regioselectivity, and limited cofactor requirements [1,2,5,6,7,8,9].

At least three important aspects must be considered while developing an assay for PLA2. First, a suitable phospholipid substrate needs to be selected for the enzyme to act upon, as it is known that different isoforms of PLA2 can have preferences over different substrates [1,2,3,4]. Secondly, since this family of phospholipases consists of interfacial esterases, thus acting on an assembled phospholipid supramolecular structure rather than on a monomeric lipid, [10] they are expected to be impacted by the nature of the supramolecular assembly of the phospholipids in water (e.g., micelles, vesicles, etc.), determined by the individual packing parameter of the amphiphiles [11] and their molar fraction in the supramolecular assembly [11,12,13,14,15,16] Thirdly, selecting a suitable detection method that is sensitive enough to measure the enzyme activity and inhibition/activation accurately is of critical importance [9,17,18,19].

**Scheme 1:**
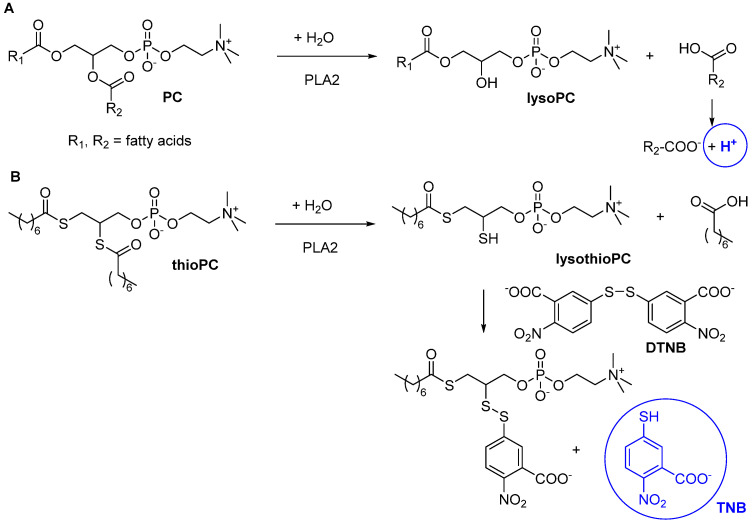
Most PLA2 assays are based either on the PLA2-mediated hydrolysis of natural phospholipids such as egg PCs (**A**), when the one quantifies the acid produced in the reaction, or on the PLA2-mediated hydrolysis of synthetic thioPCs (**B**), when the resulting lysothioPC further reacts with DTNB to yield the TNB final product, which is quantified spectrophotometrically at 412 nm.

PLA2 assays were initially developed using natural substrates collected from egg PCs (PCs containing a mixture of palmitic, stearic, oleic, linoleic, and arachidonic acids) (Scheme 1A) [20,21,22,23,24,25]. However, these assays were deemed not sensitive enough and lacked PLA2 hydrolysis quantification in real time [20,21,22,23,24,25]. Consequently, thioester-based substrate assay systems were developed and have been widely used in PLA2 assays [26,27,28,29]. In this context, Aarsman et al. showed that replacing the ester group with a thioester one at the sn-2 position of the phospholipids yields thiolipids that can be processed faster by the PLA2 enzyme, as the C–S bond is significantly weaker than the C–O bond (Scheme 1B) [30]. The study showed that PLA2s can hydrolyze thioester compounds two to three times faster than their regular ester analogs, releasing products containing a free thiol group (e.g., lysothioPC in Scheme 1B). This thiol group subsequently reacts with 5,5′-dithiobis (2-nitrobenzoic acid) (DTNB, Ellman’s reagent), yielding the chromogenic product 5-thio-2-nitrobenzoic acid, which can be traced spectrophotometrically via its absorbance at 412 nm, [27,28] translating into an improved, sensitive assay (Scheme 1B) [30]. Although very sensitive and suitable for assessing PLA2 inhibitors in medicinal chemistry studies [27,28], these assays cannot be used to assess the interaction of PLA2s with natural phospholipids in their supramolecular assemblies used for drug and nucleic acid delivery or with synthetic glycerol-based lipids used for similar purposes [14,16,31,32,33].

Consequently, the aim of this study was to develop an assay for PLA2 using a pure POPC-based natural substrate in various supramolecular assemblies by measuring the pH change during the hydrolytic reaction with a pH-sensitive indicator dye, spectrophotometrically. This assay would provide a common ground for assessing PLA2 activity on different phospholipid self-assemblies (liposomes, mixed micelles, etc.) and for determining the kinetic parameters of PLA2 while processing phospholipids within those supramolecular assemblies, with relevance for their interaction with natural assemblies and with different drug and nucleic acid delivery systems containing glycerol-based natural and synthetic amphiphiles.

## 2. Results and Discussion

### 2.1. Preparation and Characterization of POPC Liposomes and Mixed Micelles

**Figure 1:**
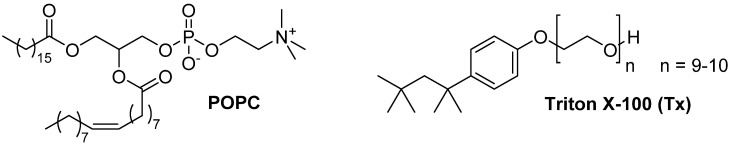
Chemical structures of POPC and of Triton X-100. POPC is a pure phospholipid, fluid at room temperature (Tm = −2 °C), and Triton X100 (Tx) is an amphiphile that has well-defined physicochemical properties and a low CMC of 0.3 mM, which facilitates the formation of supramolecular structures, including mixed micelles with natural phospholipids, such as POPC and congeners.

The natural phospholipid 3-palmitoyl-2-oleoyl-sn-glycero-1-phosphocholine (POPC, Figure 1) is considered a good choice for developing assay methods for PLA2s since most lipid-based delivery systems are based on natural substrates [34]. Natural PCs are a common component of the cell membrane and therefore perfectly biocompatible, which makes them preferred components in drug and nucleic acid delivery systems [14,31], including the two COVID-19 vaccines [16]. POPC is a double acyl-chained long lipid, fluid at room temperature (Tm = −2 °C), with a packing parameter between 0.5 and 1, which helps it to form bilayer vesicles with a hydrophilic interior and a hydrophobic intra-bilayer space [11,31,35].

POPC is also suitable for forming mixed micelles, as it can be readily mixed with amphiphiles such as Triton X-100, forming these types of supramolecular assemblies [36,37]. Triton X-100 is a nonionic surfactant with well-defined physicochemical properties (Figure 1) [37]. It has a hydrophilic polyethylene oxide chain (9–10 units in length) attached to a 4-(1,1,3,3-tetramethylbutyl)-phenyl hydrophobic group, with a molecular weight M_n_ ~625 g/mol. Due to its nonionic nature and low packing parameter, Triton X-100 (Tx) has been used as a common surfactant in micellar formulations. It does not introduce any charged species into the assay system and has a low critical micellar concentration (CMC) of 0.3 mM, which helps in forming micellar structures at a relatively lower concentration [36,37,38]. Oppositely, anionic amphiphiles used in PLA2 assays such as deoxycholate have high CMC (2 mM), interact with Ca^2+^ ions in the assay buffer, and have an effect on the pH and the ionic strength of the buffer system [39]. For Tx/POPC mixed micelles, we have selected molar ratios of Tx:POPC of 2:1, 1:1, 1:2, and 1:6.67 molar ratios because at higher Tx:POPC molar ratios, an apparent inhibition of the enzyme was reported, attributed to a surface dilution effect, [40] and at lower molar ratios, the mixed micelles were no longer stable and unimodal [37].

The DLS size distribution of both POPC liposomes and Tx/POPC mixed micelles (Tx:POPC = 2:1, 1:1, 1:2, and 1:6.67 molar ratios), made via extrusion and sonication methods is presented in Table 1 below. The pure POPC liposomes, made through the extrusion of a hydrated POPC lipid film, were about 108 nm in size (main peak), with a PDI value of 0.1 (Table 1). The extrusion method has reasonable control over the sizes of the liposome formulation, generating a unimodal distribution, as shown by DLS data (Table 1). The mixed micelles POPC/Tx, made by the sonication method, displayed a broad particle size distribution, ranging from 30 nm (for Tx:POPC 1:6.67 molar ratio) to 50 nm for Tx:POPC 1:6.67 molar ratio (the main peak), heavily depending on the molar ratio of the components. The polydispersity index (PDI) values, reflecting the polydispersity of the formulations, were higher than in the case of liposomes, which was typical for micelles, as well as for self-assemblies generated by the sonication method (Table 1). The mixed micelles Tx:POPC 1:6.67 molar ratio could be considered the richest in POPC micelles that were still unimodal (main peak > 90% by volume measurement in DLS). Raising the amount of POPC further in the mixed micelles yielded multimodal systems.

### 2.2. Preparation of Standard Absorption Curve of Bromothymol Blue (BTB)

Bromothymol blue (BTB) is a pH indicator that is frequently used for detecting the changes in the pH of a solution [41]. BTB is a weak acid that can be protonated and deprotonated by small changes in pH. Therefore, it has a very good sensitivity around pH = 7, which is particularly advantageous, as it allows facile monitoring of PLA2 activity at a physiologic pH of 7.4 [41,42,43]. When PLA2 hydrolyzes phospholipid substrates, it generates lysophospholipids and fatty acids. Fatty acids ionize in aqueous media, generating a pH change that can be detected and quantified using BTB [9,44].

**Figure 2:**
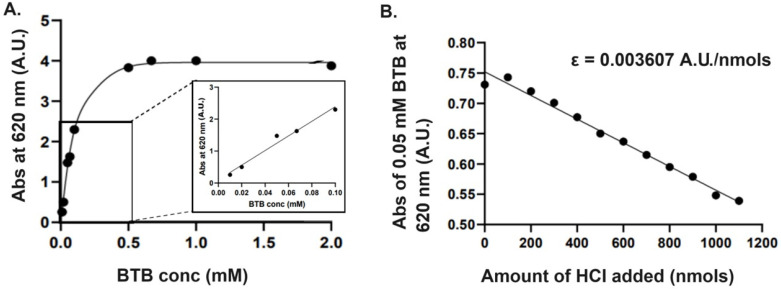
(**A**) Absorbance of BTB solution at 620 nm for BTB solutions with concentrations ranging from 0.01 mM to 2 mM and from 0.01 mM to 0.1 mM (linear dependence, insert). (**B**) Absorbance of BTB (0.05 mM) while adding HCl (100 nmols increments) to mimic the PLA2 hydrolysis reaction, revealing the maximum sensitivity of the assay.

The absorbance of the BTB solutions as a function of BTB concentration was determined and is shown in Figure 2A below. One can notice that the BTB absorbance increases linearly with the increase in its concentration up to 0.1 mM (Figure 2A and insert), reaching a plateau from 0.5 mM upwards.

Therefore, the working concentration for BTB was selected to be 0.05 mM, within the linear range, and the indicator was titrated with aliquots of HCl (100 nmols) to validate the linearity of its response to protons at A = 620 nm (Figure 2B). Data from Figure 2B confirms the linear response of BTB to acid, with a maximum sensitivity of 0.003607 A.U./nmol H^+^.

### 2.3. Effect of CaCl_2_ on sPLA2 Activity

**Figure 3:**
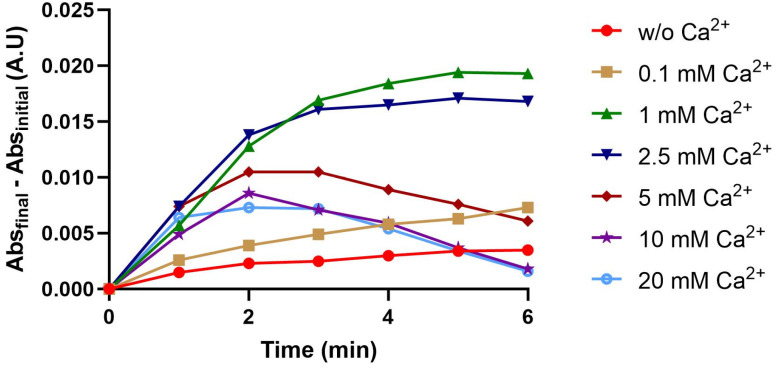
Effect of Ca^2+^ concentration on sPLA2 IIA activity, assessed via BTB (0.05 mM) using POPC liposomes (25 µM) as the substrate and albumin (1 mg/mL) as the receiving phase for fatty acids produced in the ester hydrolysis, at 37 °C. Lines connecting the points are a guide for the eye.

Ca^2+^ ion is a cofactor for sPLA2 (secreted PLA2—IIA and IB isozyme) enzymes and is required for binding of the phospholipid in the active site of the enzyme and for catalysis, with the mechanism being discussed in detail [1,2,3]. Dennis et al. showed that sPLA2 activity increased with increasing Ca^2+^ concentration until [Ca^2+^] = 10 mM, followed by a drop in activity at higher concentrations [37]. However, when we assessed the sPLA2 activity (isozyme IIA) in a system containing POPC liposomes (25 µM), albumin (as the receiving phase for fatty acids produced in the hydrolysis process, 1 mg/mL), and BTB (0.05 mM) at 37 °C while increasing concentrations of Ca^2+^ from 0 to 20 mM, we found the highest sPLA2 IIA activity within the range of 1–2.5 mM of Ca^2+^ ion (Figure 3).

Increasing the [Ca^2+^] concentration above 2.5 mM (5, 10, 20 mM) was not beneficial, after an initial activity increase, and the processivity of the sPLA IIA decreased in time, probably due to the association of resulting fatty acids with excess Ca^2+^ ions and the formation of insoluble fatty acid complexes, with detrimental effects on enzyme activity (Figure 3). Interestingly, the physiological concentration of Ca^2+^ ion in blood and extracellular fluid is between 2.1 and 2.7 mM or 8.5 and 10.5 mg/dL [45], which suggests that the enzyme has evolved to have the highest processing capacity around the physiological concentration of Ca^2+^ ions.

### 2.4. Effect of pH on PLA2 Activity

**Figure 4:**
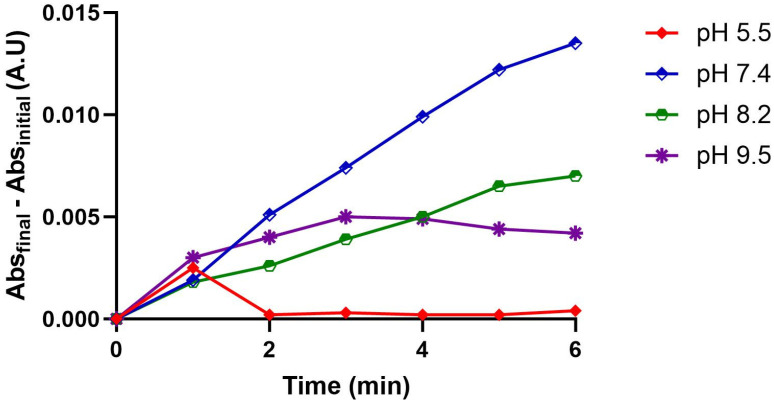
Effect of pH on PLA2 activity on POPC liposomes (25 µM), determined with buffers ranging from pH 5.2 to 9.5, in the presence of 1 mM Ca^2+^, 0.05 mM BTB, and 1 mg/mL albumin, at 37 °C. Lines connecting the points are a guide for the eye.

pH is another important factor for PLA2 activity [1,2,3]. PLA2 works optimally around the physiologic pH of 7.4. However, in different disease conditions, the local pH may vary, affecting PLA2 activity [1,2,3]. Consequently, we varied the pH of the reaction mixture while keeping all the other assay components constant (POPC liposomes 25 µM, 0.05 mM BTB, 1 mM Ca^2+^), at 37 °C (Figure 4). Data from Figure 4 revealed that the peak of PLA2 activity is at pH 7.4, decreasing significantly when the pH is either reduced (pH = 5.5, encountered in endosomes) or raised to 8.2 and 9.5, respectively (Figure 4).

### 2.5. Comparison of PLA2 IIA Kinetics on POPC-Based Self-Assemblies (Liposomes vs. Mixed Micelles)

**Figure 5:**
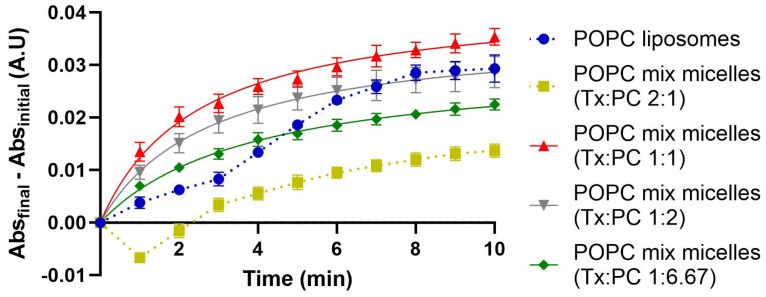
Comparison of substrate processivity by PLA2 based on different POPC self-assemblies (liposomes vs. mixed micelles) at the same overall POPC substrate concentration (25 µM).

When PLA2 acts on phospholipid self-assemblies (liposome/mixed micelles) rather than on individual substrate molecules, the rate of hydrolysis depends not only on the substrate concentration of the phospholipids but also on the average surface area covered by phospholipids on the self-assembly that is accessed by the enzyme, a phenomenon called the “surface dilution kinetics model” [37,40,46,47]. This phenomenon could also be used to discuss the preliminary PLA2 kinetics data on liposomes and mixed micelles, as shown in Figure 5 below. Data from Figure 5, generated using the previously optimized assay conditions (0.05 mM BTB, 1 mM Ca^2+^, 1 mg/mL albumin, at 37 °C, and pH = 7.4), at equimolar concentrations of POPC substrate (25 µM) in both the liposomal and in the Tx mixed micelle formulations, were used to compare the PLA2 activity on these supramolecular assemblies. Thus, PLA2 IIA processing of POPC from liposomal assemblies (the blue line in Figure 5) occurs initially slowly. After about 3 min, the processivity of the enzyme increased significantly. One can imagine that the POPC phospholipids in the liposomes are arranged in a bilayer and that PLA2 docks on the outer layer of the liposomes and starts hydrolyzing the phospholipids there. The enzyme initially does not have access to the inner leaflet pool of lipids within liposomes. Once lysolipids are generated due to PLA2 activity, the bilayer (liposomal) assemblies gradually shift towards micellar form, as the packing parameter of the lysophospholipids is higher than the packing parameter of POPC, thus increasing the enzyme access to the substrates. Mixed micelles, on the other hand, have all the phospholipids distributed on a single surface, along with the Triton X-100. Thus, the enzyme has full access to the entire pool of substrate from the beginning. Data from Figure 5 show that three of the four mixed micelles formulations (Tx:PC 1:1, 1:2, 1:6.67) have higher initial velocity for enzyme-substrate processivity as compared with the liposomal assemblies. However, in the case of Tx:PC 2:1, an initial apparent drop in substrate processivity can be observed, probably due to the inability of enzymes to dock on the surface of the mixed micelles due to a high concentration of Triton X-100, which interferes with the PLA2 activity, as reported previously [37,40,46,47]. The highest processivity for PLA2 IIA is observed when POPC is mixed with Triton X-100 in mixed micelles at an equimolar ratio (red curve in Figure 5), followed by the 1:2 ratio (gray curve), 1:6.67 ratio (green curve), and 2:1 ratio (light green curve), indicating a strong dependence of the processivity of the PLA2 IIA on the molar fraction of POPC in the mixed micelles (Figure 5).

### 2.6. Comparison of PLA2 IIA and IB Assay Kinetics with POPC-Based Liposomal Vesicles and Mixed Micelles

**Figure 6:**
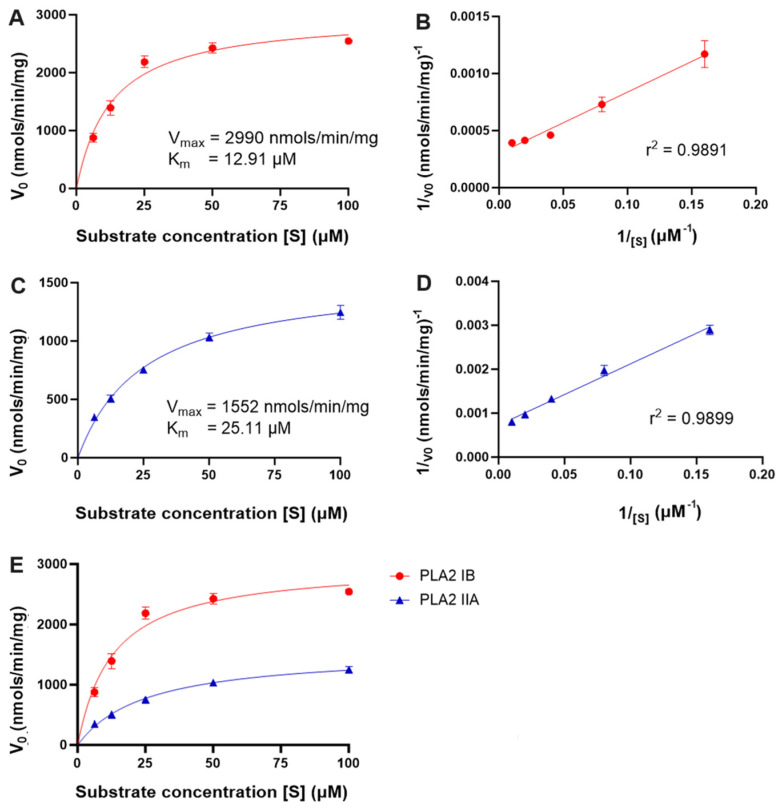
Enzyme kinetics of PLA2 IB and PLA2 IIA using POPC-based liposomes: Michaelis–Menten kinetics and double reciprocal Lineweaver–Burke plots for PLA2 IB (**A**,**B**) and PLA2 IIA (**C**,**D**), together with a direct comparison of the kinetics of the two isozymes (**E**).

A comparative PLA2 assay kinetic study was conducted using two different isoforms of sPLA2, namely IB and IIA, using POPC in liposomal form. Five different POPC liposome substrate concentrations were used to determine the initial velocity of each PLA2 (IB and IIA) as a function of substrate concentration. Kinetic data for both the isozymes fitted in the Michaelis–Menten equation provided a V_max_ of 2990 nmols/min/mg and 1552 nmols/min/mg for PLA2-IB and PLA2-IIA, respectively. The Km values for the substrate were 12.91 μM and 25.11 μM, respectively (Figure 6A–D, also showing the linear Lineweaver–Burke plot for both isozymes). The specific activity of both isozymes was superimposed on Figure 6E, clearly showing that PLA2-IB was able to process more substrate per unit of time compared to PLA2-IIA. This also confirms the substrate preference of PLA2 isozymes, as it is known that PLA2-IB has a higher preference for zwitterionic PCs than PLA2-IIA [1,3].

**Figure 7:**
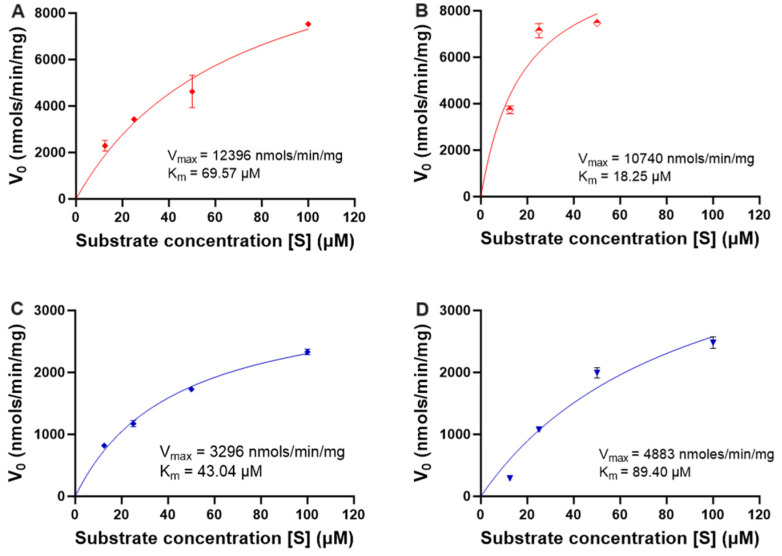
Comparative enzyme kinetics of PLA2 IB on POPC-based mixed micelles Tx:POPC 1:1 (**A**) and Tx:POPC 1:2 (**B**), as well as for PLA2 IIA on POPC-based mixed micelles Tx:POPC 1:1 (**C**) and Tx:POPC 1:2 (**D**).

The kinetic data of PLA2 IB and PLA2 IIA on Tx/POPC mixed micelles formulations as a substrate at two molar ratios (Tx:PC 1:1 and 1:2) are shown in Figure 7. The V_max_ values of PLA2-IB on Tx:PC 1:1 and 1:2 mixed micelles were determined to be 12,396 nmols/min/mg and 10,740 nmols/min/mg with Km values of 69.57 µM and 18.69 µM, respectively (Figure 7A,B). Similarly, the V_max_ values for PLA2-IIA were found to be 3296 nmols/min/mg and 4883 nmols/min/mg with Km values of 43.04 µM and 89.40 µM for mixed micelles Tx:PC 1:1 and 1:2, respectively (Figure 7C,D). By comparing the data of Figure 6 and Figure 7, one can clearly observe that each isozyme was impacted by the nature of the phospholipid’s assemblies and that both isozymes processed more substrate from the mixed micelles assemblies as compared with the liposomal assemblies. However, POPC liposomes displayed lower Km values for both isozymes, indicating that both PLA2 isozymes have a higher affinity for POPC liposomes as compared to Tx/POPC mixed micelles.

### 2.7. Inhibition Study of PLA2 IIA Using Varespladib

**Figure 8:**
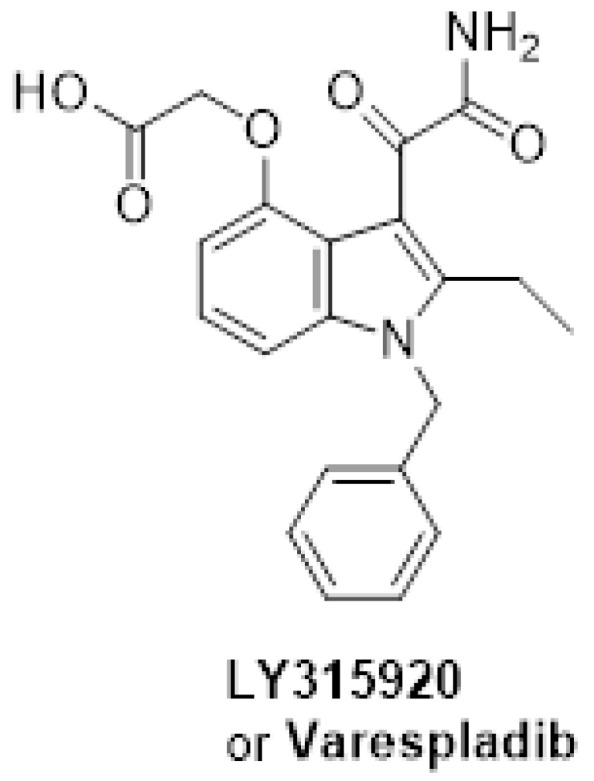
Chemical structure of varespladib—an indole class PLA2-IIA-selective inhibitor [2].

We have also investigated the inhibition of PLA2 using the newly developed assay to further evaluate its usefulness and sensitivity. Varespladib is a potent inhibitor of PLA2 selective for group IIA, developed by Eli Lilly (Figure 8) [2,27,28,29]. The compound was tested using the thio assay developed by Dennis et al. and showed an IC_50_ value of 9 nM [2,26,29].

**Figure 9:**
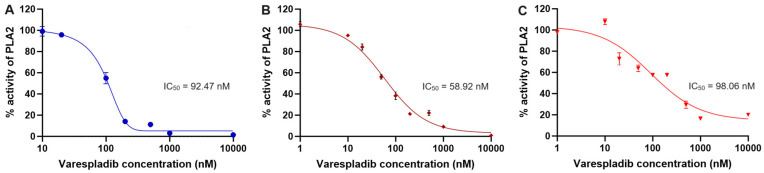
Determination of IC_50_ of varespladib using POPC-based liposomes (**A**) and mixed micelles Tx:POPC 1:1 (**B**) and Tx:POPC 1:2 (**C**).

The IC_50_ value of varespladib was determined using our assay by varying its concentration from 1 nM to 10 µM in the PLA2 assay while keeping all the other assay components unchanged (Figure 9). Data from Figure 9 revealed an IC_50_ value for varespladib of 104.0 nM for liposomal assemblies and 58.92 nM and 98.06 nM for mixed micelles of Tx:PC 1:1 and 1:2, respectively. Importantly, varespladib was able to inhibit PLA2 activity more than 97% when assessed with the POPC substrate as liposomes and mixed micelle at Tx:PC 1:1. However, for mixed micelles Tx:PC 1:2, the inhibition was not higher than 85% of the total enzyme activity. The IC_50_ values for varespladib probably differ because of the nature of the substrate and how it self-assembles at different ratios of Triton X-100. Mention must be made that the IC_50_ value also varies within the traditional thio-PC assays because of the differences in the free fatty acid leaving the site after hydrolysis of the thioester by the enzyme [2,26,29].

**Figure 10:**
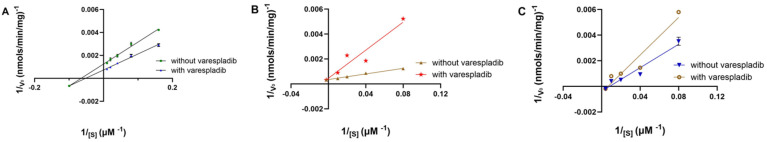
Comparison of PLA2 IIA inhibition mechanism of varespladib in POPC liposomes (**A**) and mixed micelles Tx:POPC 1:1 (**B**) and Tx:POPC 1:2 (**C**).

A comparison of the inhibition mechanism of varespladib in liposomes and mixed micelles is shown in Figure 10. Interestingly, varespladib had different mechanisms of inhibition on different POPC assemblies, revealing the importance of the interfacial interactions in PLA2 inhibition. In Figure 10, we used varespladib at a concentration of 100 nM to measure PLA2 activity at different substrate concentrations. The Lineweaver–Burke plot was constructed in all cases and compared with similar plots obtained without an inhibitor. The intersection of the two data sets indicated that varespladib acted in a mixed inhibition fashion in the case of liposomes (the two lines met at the third quadrant, Figure 10A). In contrast, in the case of mixed micelles (for both Tx:POPC 1:1 and 1:2), the line met at or near the *y*-axis, indicating that varespladib was acting as a competitive inhibitor against PLA2 (Figure 10B,C).

## 3. Materials and Methods

### 3.1. Materials

The lipid POPC (850457P) was obtained from Avanti Polar Lipids, Inc. (Alabaster, AL, USA). CaCl_2_ was purchased from Aldrich Chemical Company, Inc. (St. Louis, MO, USA), bromothymol blue (A17746) was purchased from Alfa Aesar (Themo Fischer Scientific, Ward Hill, MA, USA). Albumin, bovine (0332), was obtained from VWR Inc (Solon, OH, USA). HEPES (4-(2-hydroxyethyl)-1-piperazineethanesulfonic acid) (0511) was obtained from AMRESCO (Allentown, PA, USA). Triton X-100 was purchased from Fischer Scientific (Waltham, MA, USA). PLA2-IIA (source—Crotalus adamanteus) was obtained from Worthington Biochemical Corp (Lakewood, NJ, USA). PLA2-IB (source—Porcine pancreas) was obtained from Sigma Aldrich (St Louis, MO, USA). Varespladib (LY315920) was purchased from Cayman Chemical Company (Ann Arbor, MI, USA).

### 3.2. Methods

#### 3.2.1. Preparation of Buffers and Stock Solutions

The 4-(2-Hydroxyethyl)-1-piperazineethanesulfonic acid (HEPES) buffer saline (HBS) 1X was prepared by dissolving 6 g of HEPES-free acid, 4 g of NaCl, and 0.271 g of Na_2_HPO_4_^.^12H_2_O in a volume of 1000 mL of DI water. The pH of the buffer was adjusted to 7.4 by adding either concentrated NaOH or HCl. The concentration of this buffer was 25.17 mM HEPES, 68.44 mM NaCl, and 0.76 mM Na_2_HPO_4_, with pH = 7.4.

A CaCl_2_ stock solution of 100 mM was prepared by dissolving 11.1 mg of dry CaCl_2_ powder in 1 mL HBS (1X). The bovine serum albumin (BSA) stock solution of 100 mg/mL was prepared by dissolving 110 mg of BSA powder in 1100 μL of HBS (1X), and the bromothymol blue (BTB) stock solution of 5 mM in ethanol:HBS (1:1) was prepared by dissolving 12.51 mg of BTB powder in 2 mL of ethanol and then adding 2 mL of HBS (1X).

Phospholipase A2 IB (porcine pancreas, molecular weight 13.9 kDa) was stored as an ammonium sulfate suspension at a concentration of 2.59 mg/mL. The strength of the enzyme was about 1618 U/mg. According to the supplier, 1 U enzyme will hydrolyze 1.0 µmol soybean L-α-phosphatidylcholine to L-α-lysophosphatidylcholine and a fatty acid per min at pH 8.0 and at 37 °C. The working stock solution was prepared by diluting 19 μL of the main stock with HBS 1X to a final volume of 1000 μL, which made the concentration of the working stock solution 49.44 μg/mL (3.54 μM or 80 U/mL).

Phospholipase A2 IIA (Crotalus adamanteus, molecular weight 30 kDa) was stored as dried powder in an amber vial as a 1 mg pack with a strength of 460 U/mg. According to the supplier, 1 U enzyme will hydrolyze 1.0 µmol soybean lecithin per minute at pH 8.9 and at 25 °C. The whole content was dissolved in 5 mL HBS 1X, making the concentration of the main stock 0.2 mg/mL (6.67 μM). The strength of the enzyme was 92 U/mL. A working stock solution was made by taking 100 μL of the main stock and adding 15 μL of HBS 1X to adjust the strength of the enzyme to 80 U/mL. The concentration of the working stock was 0.174 mg/mL (5.8 μM).

#### 3.2.2. Preparation of 1.0 mM POPC Liposomes via Extrusion Method

The liposomes were prepared by the thin film hydration technique followed by the extrusion method used by the authors and others [33,48,49,50]. Briefly, a 3 mM stock solution of POPC lipids was prepared by dissolving 6.84 mg of POPC dry powder in 3 mL of chloroform:methanol (2:1) solvent mixture. A volume of 666.7 μL of POPC solution from 3 mM stock was diluted with chloroform:methanol (2:1) solvent mixture to a final volume of 2000 μL in a clean vial, making a 1 mM POPC solution. The solvent was evaporated using a rotary evaporator, forming a thin film of lipids on the wall of the vial. The vial was kept under vacuum at 40 °C for an additional 2 h to ensure that all the traces of solvent were removed. The dried lipid film was hydrated with 2 mL of HBS 1X and then freeze-thawed 5 times using acetone (−65 °C) and water (75 °C) bath. Subsequently, the hydrated lipid suspension was loaded and pre-heated at 30 °C for 20 min and extruded using a mini extruder (Avanti Polar Lipids Inc., Birmingham, AL, USA), by passing them through a 0.1 µm membrane filter (Avanti Polar Lipids Inc., Birmingham, AL, USA). The hydrodynamic radius of the liposomes and the polydispersity index were measured using a dynamic light scattering technique using a ZetaSizer Nano (Malvern Panalytical, Malvern, UK), using a standard 12 mm o.d. square glass cell with square aperture (e.g., PCS1115, Malvern Panalytical, Malvern, UK) and using the “volume” reading of the instrument software. The liposomes were stored at 4 °C for further experiments.

#### 3.2.3. Preparation of POPC Mixed Micelles with Triton X-100 via Sonication Method

The mixed micelles were prepared by the thin film hydration technique followed by the sonication method [36]. Briefly, a volume of 333.33 μL of POPC solution from the 3 mM stock solution in the chloroform:methanol (2:1) solvent mixture was diluted with the same solvent mixture to a final volume of 1 mL, making a final lipid concentration of 1 mM, in 4 different clean vials. The solvent was evaporated using a rotary evaporator, forming thin films of lipids on the walls of the vials. The vials were kept under vacuum at 40 °C for 2 h to ensure that all the traces of solvents were removed. Four different mixed micelles formulations were prepared using Triton X-100 (Tx) and POPC in molar ratios of 2:1, 1:1, 1:2, and 1:6.67, respectively. The mixed micelles Tx:PC 2:1, 1:1, 1:2, and 1:6.67 were prepared by adding either 1000 μL, 500 μL, 250 μL, or 150 μL of 2 mM Triton X-100 solution in HBS 1X over the dried lipid-containing vial and adjusting the volume to a final volume of 1000 μL with HBS 1X. All the formulations were prepared by adding the Triton X-100 solution first while vortexing the vial simultaneously and then gradually adding the HBS 1X buffer. The vials were sonicated for 30 min, and DLS readings were performed as indicated above to measure the mixed micelles size distribution.

#### 3.2.4. Preparation of Standard Curve Using Bromothymol Blue (BTB)

A standard curve of bromothymol blue (BTB) was prepared from a 5 mM stock solution of BTB in methanol:HBS (1:1). The stock solution was serially diluted from 5 mM to 0.01 mM, and absorbance readings were taken at 620 nm. The absorbance vs. concentration curve for BTB was plotted, and the linearity of the curve was found to be in the range of 0.01–0.1 mM BTB concentration. Subsequently, a working concentration for the indicator was selected at 0.05 mM BTB solution.

The sensitivity of the assay was assessed by adding known amounts of HCl in the working concentration of BTB. Thus, a second standard curve was prepared by sequential addition of HCl aliquots (10 μL of 1M HCl, 100 nmols) in 1 mL of 0.05 mM BTB solution and recording the absorbance at 620 nm after each addition. The sensitivity of the assay was determined by averaging three y/x values.

#### 3.2.5. Effect of Ca^2+^ Ion on PLA2 Activity

The effect of Ca^2+^ ion on PLA2 activity was studied using a standard PLA2 assay with modification [26], where all the components of the assay were kept unchanged and with a varying concentration of CaCl_2_. Briefly, in a glass cuvette, POPC liposomes, BSA (bovine serum albumin), and BTB were taken at concentrations of 25 μM, 1 mg/mL, and 0.05 mM, respectively. CaCl_2_ was added at 7 different concentrations ranging from 0 mM to 20.0 mM. The mixture volume was filled up to 995 μL with HBS 1X at a pH of 7.4. The cuvette was equilibrated at 37 °C for 10 min. Finally, 5 μL of the PLA2-IIA enzyme was added at a strength of 400 mU/mL to the total volume of 1000 μL. Absorbance was recorded at 620 nm every min for up to 10 min. All the absorbance values were normalized to point 0, considering the time of enzyme addition as the initial time of the reaction.

#### 3.2.6. Effect of pH on PLA2 Activity

The effect of pH was studied using a method similar to the Ca^2+^ ion study. The effect of pH on PLA2 activity was studied using a standard PLA2 assay with modification [26], varying the pH of the buffer while keeping the rest of the assay component unchanged. Briefly, in a glass cuvette, POPC liposomes, CaCl_2_, BSA, and BTB were taken at concentrations of 25 μM, 1 mM, 1 mg/mL, and 0.05 μM, respectively. The mixture volume was filled up to 995 μL with HBS with four different pH levels. These four different buffer systems were prepared using four different pH levels, which were adjusted with an HCl and NaOH concentrated solution. The change in PLA2-IIA activity was compared.

#### 3.2.7. Assay Method for PLA2 Kinetic Study Using POPC-Based Liposomes and Mixed Micelles

The assay protocol was developed, optimizing the assay parameters following guidelines from the published literature [17,26,44]. All the experiments were done in a glass cuvette, at a final reaction mixture volume of 1 mL. Absorbance was recorded at 620 nm with a SpectraMax M2 instrument. All the components were mixed in the following order in HBS 1X buffer: 10 μL of 100 mg/mL BSA, 10 μL of 100 mM CaCl_2_, and 10 μL of 5 mM BTB, making a final concentration of 1 mg/mL BSA, 1 mM Ca^2+^, and 0.05 mM BTB, respectively. The POPC substrate (liposomes or mixed micelles) was added separately in four different concentrations (6.25–100 μM) into four sets of experiments. The final volume of the reaction mixture was adjusted to 995 μL with the HEPES buffer prior to enzyme addition. The cuvette was equilibrated for 10 min at 37 °C, and then a volume of 5 μL of either PLA2 IB or IIA from their respective stock solution was added to generate final concentrations of PLA2-IB and PLA2-IIA of 17.62 nM and 29 nM, respectively, both having equal enzymatic strength of 400 mU/mL in the reaction volume of 1000 μL. The reaction mixture was mixed for 20 s, and then readings were taken every minute for up to 10 min. Each set of experiments was done in triplicate.

#### 3.2.8. PLA2 Inhibition Study with the Developed Assay Using a Standard Inhibitor Varespladib

A 10 mM varespladib stock solution was prepared by dissolving 1.1 mg of varespladib dry powder in 289.2 μL of DMSO. The stock solution was diluted 1000-fold using HBS to a working inhibitor concentration of 10 μM, reducing the DMSO concentration to 0.1%. The dilution was done in two steps: First, 10 μL of 10 mM varespladib stock in DMSO was diluted with 990 μL of HBS to yield a 100 μM stock. Then, 100 μL of 100 μM stock of varespladib was diluted with 900 μL of HBS to a final concentration of 10 μM. The inhibition assay was conducted using the PLA2 kinetic assay protocol that had been developed earlier, mixed the components as reported above to generate a final concentration of 1 mg/mL of BSA, 1 mM CaCl_2_, and 0.05 mM BTB in HBS. For determining the IC_50_ value, varespladib was added at a range of 1 nM–10 μM concentration with POPC substrate (liposomes or mix micelles, both at a concentration of 25 μM). For determining the inhibition kinetics, varespladib was used at 100 nM concentration with POPC substrate at four different concentrations (12.5 μM, 25 μM, 50 μM, 100 μM) for each set of experiments. The volume of the reaction mixture was adjusted to 995 μL before adding the enzyme. The reaction mixture was equilibrated at 37 °C for 10 min, and 5 μL of 5.8 μM of sPLA2 IIA was added to the mixture, with the absorbance recorded at 620 nm every min for up to 10 min. Each set of experiments was done in triplicate. The initial velocity of the reaction was measured by calculating the slope of the first 5 min and then dividing it by the absorptivity constant. A typical Michaelis–Menten enzyme kinetics graph was produced when the initial velocity was plotted as a function of substrate concentration. The curve was linearized using the Lineweaver–Burke double reciprocal plots.

## 4. Conclusions

In this study, we developed an assay for PLA2s using the pH-sensitive indicator dye BTB, which allowed tracking of the natural phospholipid substrate POPC processivity by the enzymes in real time, whether in liposomal form or in mixed micelles with Triton X-100 at different molar ratios. The interfacial nature of these isozymes was clearly revealed, together with the significant impact of the substrate aggregation form (liposomes, mixed micelles, and their composition) on the enzymatic activity. This assay is suitable for assessing the activity of different PLA2 isozymes and allows the determination of kinetic parameters of these isozymes and their inhibition with great accuracy using UV-VIS spectrophotometry for screening of enzymes, substrates, and inhibitors.

## Figures and Tables

**Table 1:** Size distribution of POPC-based liposomes and mix micelles.

Formulations	Z.avg (nm)	Main Peak (nm)	PDI	%Volume
POPC pure (liposomal)	113.3	108.4	0.10	100%
Tx:POPC 2:1 mixed micelles	70.0	50.3	0.22	>95%
Tx:POPC 1:1 mixed micelles	63.1	32.0	0.37	>98%
Tx:POPC 1:2 mixed micelles	63.7	33.4	0.41	>98%
Tx:POPC 1:6.67 mixed micelles	108.8	30.4	0.50	>90%

## Data Availability

The data presented in this study are available in the article. Additional data are available from the corresponding author upon request.
